# Secondary Amyloidosis of the Urinary Bladder Presenting As Urinary Retention

**DOI:** 10.7759/cureus.84096

**Published:** 2025-05-14

**Authors:** Wyatt MacNevin, Ainsley Bethune, Cheng Wang, Ashley Cox

**Affiliations:** 1 Department of Urology, Dalhousie University, Halifax, CAN; 2 Department of Family Medicine, Dalhousie University, Halifax, CAN; 3 Department of Pathology, Dalhousie University, Halifax, CAN

**Keywords:** bladder amyloidosis, cystoscopy, lower urinary tract dysfunction, secondary amyloidosis, urinary retention

## Abstract

Bladder amyloidosis is a rare condition of insoluble extracellular protein deposition in the urinary bladder arising as a complication of underlying inflammation and immune dyscrasia. Diagnosis of bladder amyloidosis is done through cystoscopy and evidence of amyloid deposition on pathologic evaluation. Here, we present a case of secondary bladder amyloidosis presenting with acute urinary retention. After ruling out bladder malignancy, the patient was managed with suprapubic catheterization after failing beta-3 agonist therapy and clean intermittent catheterization.

## Introduction

Bladder amyloidosis is a rare entity with fewer than 200 documented reports in the literature. Bladder amyloidosis often presents with gross hematuria (GH), dysuria, and/or acute urinary retention [1-3]. Non-specific signs of mucosal erythema and protuberant papillary-appearing lesions are commonly seen on cystoscopy, which must be discerned from malignancy [4,5].

Characterization and diagnosis of bladder amyloidosis is done through biopsy and histological evidence of amyloid deposition, reactive to Congo red staining and apple-green birefringence upon exposure to polarized light [6,7]. Due to the rarity of bladder amyloidosis, there is a scarcity of literature detailing effective management options [6]. It is further complicated as bladder amyloidosis is often associated with neurological lower urinary tract dysfunction (LUTD) with significant implications on patient quality of life and renal preservation [3]. Therefore, descriptive case presentations can provide insight into the optimal management of this condition.

Here, with patient consent, we present a case of secondary bladder amyloidosis in a 62-year-old male who developed acute urinary retention with cystoscopic findings of a bladder lesion. The lesion was initially thought to be a malignant neoplasm, but was determined to be amyloidosis after pathological analysis.

## Case presentation

A 62-year-old male with a history of localized light-chain amyloidosis and marginal zone stage IIIa lymphoma was referred to urology for acute urinary retention (AUR). The patient underwent three cycles of R-bendamustine followed by zanubrutinib after no interval radiological response. Zanubrutinib was then discontinued due to orthostatic hypotension. While receiving inpatient treatment, the patient developed AUR thought to be related to neurogenic LUTD, and Urology was consulted for evaluation.

**Figure 1:**
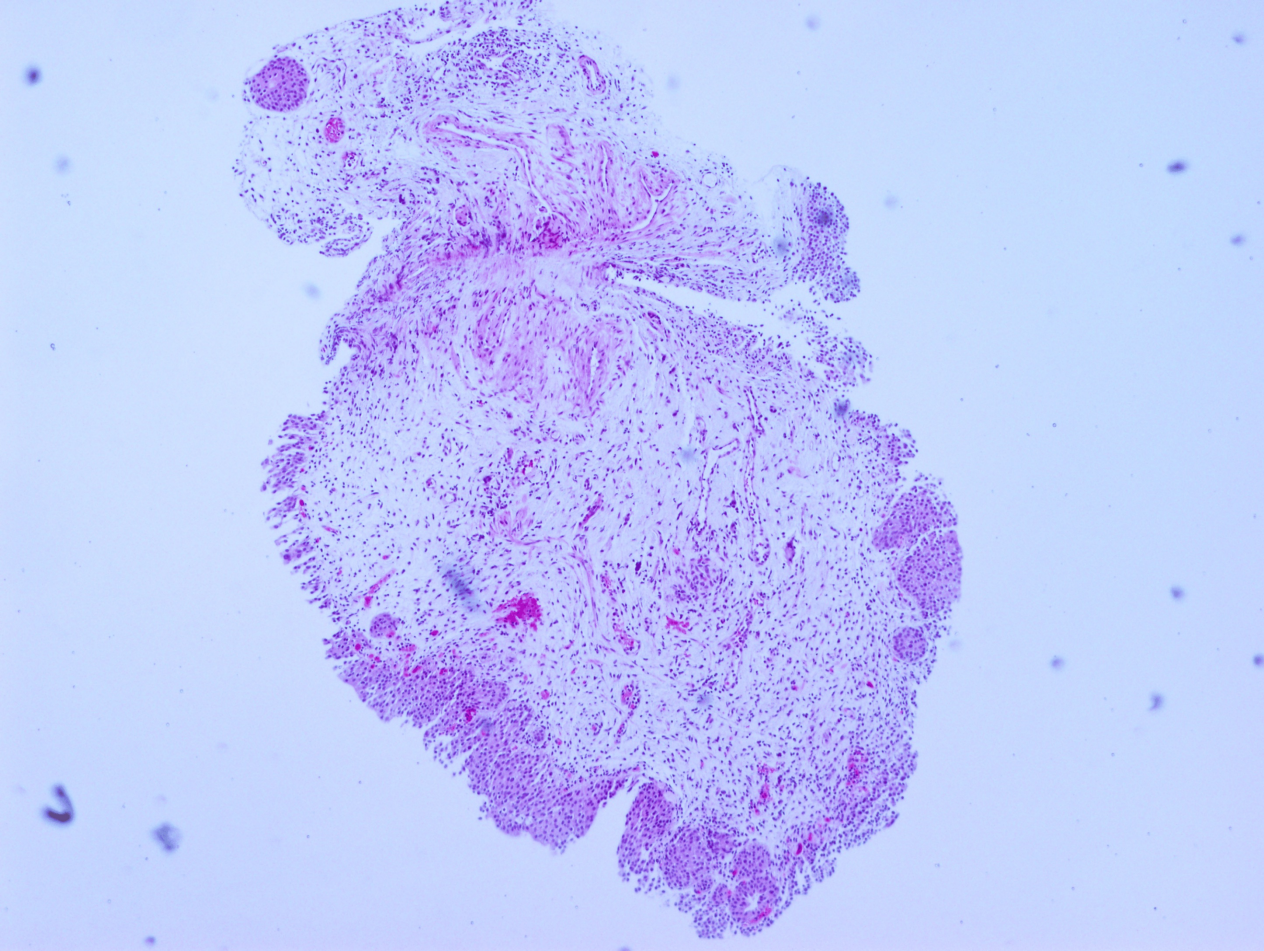
Subepithelial bladder vessels containing eosinophilic material (H&E, 4x magnification) H&E: Hematoxylin and eosin

**Figure 2:**
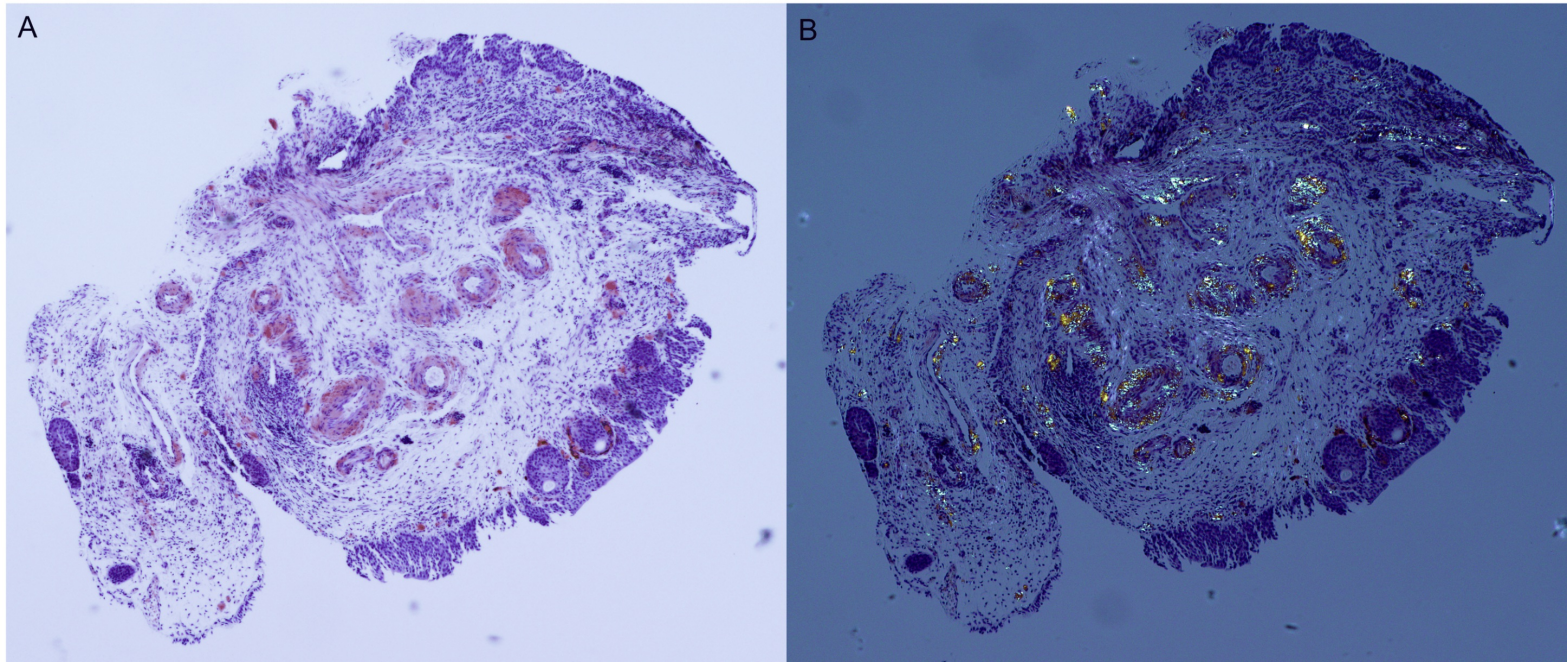
Histsopathology images of Congo red stain and apple-green birefringence A: Subepithelial bladder vessels with salmon-pink amyloid deposition (Congo red, 4x magnification), B: Visualization of apple-green birefringence (Congo red, 4x magnification, polarized light)

Cystoscopy revealed a large capacity bladder (~3L) with catheter-related mucosal inflammation and multiple papillary-appearing lesions. Urine culture demonstrated growth of coagulase-negative* Staphylococcus* and *Corynebacterium striatum*, and urine cytology was negative for high-risk urothelial carcinoma. The patient denied any history of prior AUR or LUTD, urinary tract infections, GH, benign prostatic hyperplasia, constitutional symptoms, smoking, or a personal/family history of genitourinary malignancy. The patient’s medical history was unremarkable apart from hypotension and lymphoma. The differential diagnosis included neoplasia, lymphoma, and hemorrhagic cystitis. Bladder biopsy demonstrated papillary cystitis with a background of cystitis cystica and amyloid deposition surrounding the vessels (Figure 1). The biopsied vessels of the bladder mucosa were surrounded by amorphous amyloid material, which was stained pink by the Congo red stain and demonstrated apple-green birefringence upon polarized light (Figure 2).

Urodynamic studies demonstrated detrusor overactivity and decreased detrusor compliance with a detrusor leak point pressure of 40 cm of water. The patient had a decreased bladder capacity (200 mL) and difficulty initiating a spontaneous void with a post-void residual of 140 mL. There was no hydronephrosis on imaging, and the patient’s creatinine was 66 μmol/L. The patient was started on a beta-3 agonist and instructed to perform clean-intermittent-catheterizations (CICs). Despite this approach, the patient had persistence in detrusor overactivity and incontinence and opted for an indwelling urethral catheter. After five months, the patient opted for suprapubic catheter (SPC) placement. At the four-month follow-up, the patient declined other forms of bladder augmentation or urinary diversion, such as cystectomy and ileal conduit, and chose to remain with the SPC for long-term bladder drainage. His condition was stable at the 12-month follow-up clinically and on cystoscopic exam. Despite being content with the SPC, to improve functionality, intravesical onabotulinumtoxinA may be trialed in the future to reduce his overactivity and urgency, and potentially allow for removal of the SPC and resumption of CICs for bladder management.

## Discussion

Amyloidosis can occur throughout the genitourinary tract, with bladder amyloidosis being an exceedingly rare condition. Less than 200 cases of bladder amyloidosis have been reported, contributing to the difficulty in treating this condition [7]. The clinical presentation of bladder amyloidosis most frequently includes painless GH and irritative voiding symptoms [8-10]. Less frequently, patients present with urinary retention [2,3,6,8]. Cystoscopy can pose diagnostic uncertainty for urologists, as the appearance of bladder amyloidosis can vary from papillary appearing lesions and ulcerated masses, to diffuse thickening and multifocal plaques [7]. Therefore, the differential diagnosis often includes bladder neoplasia or chronic urinary tract infections with cystitis cystica [7-10]. In this case, the patient presented with AUR with papillary-appearing lesions on cystoscopy. Of note, zanubrutinib has been linked with low incidences of GH (<10%) and AUR (<10%), although the use of zanubrutinib was stopped approximately one week before the development of this patient’s AUR. Despite this, the use of zanubrutinib may have contributed to the patient’s initial presentation. 

Due to the variance in cystoscopic appearance and clinical symptomatology and the need to exclude urothelial malignancy, the diagnosis of amyloidosis is made histologically [7]. The evidence of amyloid deposition on histology, i.e., its reactivity to Congo red staining with apple green birefringence upon exposure to polarized light, remains the gold standard for diagnosis [4,7]. Amyloid deposition can extend from the lamina propria into the muscularis propria, and some cases have reported vessel wall involvement [2,3]. The biopsied vessels in this case demonstrated amyloid deposition surrounding the bladder mucosal vessels with no vessel involvement. Depth of amyloid deposition has not been correlated with symptomatology.

Treatment of bladder amyloidosis commonly involves local transurethral resection of the amyloid lesions as monotherapy and cystoscopic follow-up. In this case, the lesions were fulgurated [2]. For patients with recurrent episodes of GH, bladder instillations with dimethyl sulfoxide (DMSO) have shown success through degradation of amyloid fibers [1,8]. Other compounds have been used in combination with local resection and DMSO, such as aluminum potassium installations post-resection to aid in reducing hematuria [1]. Cepharanthin, an anti-inflammatory and antineoplastic, and colchicine have been trialed alongside transurethral resection with unclear benefit [4,5]. Therefore, resection of the lesions in combination with DMSO remains the most effective approach to improve symptomatology [8]. Currently, the efficacy of beta-3 agonists or intravesical onabotulinumtoxinA for symptomatic management is unexplored. However, their mechanism of action can be rationalized to improve irritative voiding symptoms based on the pathophysiology of bladder amyloidosis.

## Conclusions

We described a rare case of secondary bladder amyloidosis presenting with AUR and a bladder lesion thought to be a malignant neoplasm. The patient was treated successfully with SPC after trialing CICs and a beta-3 agonist. These rare presentations can pose significant challenges concerning surgical and medical management and surveillance. Further studies are required to establish treatment recommendations for patients with bladder amyloidosis and LUTD.
